# The Interaction between *Saccharomyces cerevisiae* and Non-*Saccharomyces* Yeast during Alcoholic Fermentation Is Species and Strain Specific

**DOI:** 10.3389/fmicb.2016.00502

**Published:** 2016-04-13

**Authors:** Chunxiao Wang, Albert Mas, Braulio Esteve-Zarzoso

**Affiliations:** Departament de Bioquímica i Biotecnologia, Universitat Rovira i VirgiliTarragona, Spain

**Keywords:** contact-dependent interaction, culturability loss, excreted compounds, viable but not-culturable (VBNC), wine

## Abstract

The present study analyzes the lack of culturability of different non-*Saccharomyces* strains due to interaction with *Saccharomyces cerevisiae* during alcoholic fermentation. Interaction was followed in mixed fermentations with 1:1 inoculation of *S. cerevisiae* and ten non-*Saccharomyces* strains. *Starmerella bacillaris*, and *Torulaspora delbrueckii* indicated longer coexistence in mixed fermentations compared with *Hanseniaspora uvarum* and *Metschnikowia pulcherrima*. Strain differences in culturability and nutrient consumption (glucose, alanine, ammonium, arginine, or glutamine) were found within each species in mixed fermentation with *S. cerevisiae*. The interaction was further analyzed using cell-free supernatant from *S. cerevisiae* and synthetic media mimicking both single fermentations with *S. cerevisiae* and using mixed fermentations with the corresponding non-*Saccharomyces* species. Cell-free *S. cerevisiae* supernatants induced faster culturability loss than synthetic media corresponding to the same fermentation stage. This demonstrated that some metabolites produced by *S. cerevisiae* played the main role in the decreased culturability of the other non-*Saccharomyces* yeasts. However, changes in the concentrations of main metabolites had also an effect. Culturability differences were observed among species and strains in culture assays and thus showed distinct tolerance to *S. cerevisiae* metabolites and fermentation environment. Viability kit and recovery analyses on non-culturable cells verified the existence of viable but not-culturable status. These findings are discussed in the context of interaction between non-*Saccharomyces* and *S. cerevisiae*.

## Introduction

Spontaneous wine fermentation is driven by a succession of different yeast species. A great variety of non-*Saccharomyces* yeast species originate from grape berries and survive during the early stages of fermentation, such as species from the genera *Candida, Hanseniaspora, Lachancea, Metschnikowia, Pichia*, and *Torulaspora* (Fleet, 2003). Some species such as *Starmerella bacillaris* and *Hanseniaspora uvarum* grow to a high density (10^5^–10^7^ cells/mL) and dominate other non-*Saccharomyces* species (Wang et al., 2015b). There is a growing interest regarding the impact of non-*Saccharomyces* yeasts on the final wines, and some species are now used as fermentation starters (Jolly et al., 2014). However, fermentative *Saccharomyces cerevisiae* soon replaces non-*Saccharomyces* species to become the main or the only species present in the late stages of fermentation. Non-*Saccharomyces* strains are assumed to “die off” because these cells gradually lose their ability to form colonies on growth media, i.e., they lose the capacity to grow. The culturability loss of non-*Saccharomyces* strains has drawn widespread attention in recent years due to new findings that mention the role of excreted compounds in the interaction between *Saccharomyces* and non-*Saccharomyces* yeasts (Ciani and Comitini, 2015; Liu et al., 2015; Albergaria and Arneborg, 2016). Moreover, in a work by Branco et al. (2015), it was shown that viable but not-culturable (VBNC) status was related to interaction through excreted compounds. Therefore, as more non-conventional wine yeasts have been explored as wine starters in mixed fermentation with *S. cerevisiae* (Masneuf-Pomarede et al., 2016), studies on culturability loss of different non-*Saccharomyces* strains will help in understanding their final impact on wine quality.

The culturability loss of non-*Saccharomyces* strains at the late stages of alcoholic fermentation is a complicated phenomenon due to the multitude of factors involved. It is conventionally regarded to be related to their insufficient adaptability to environmental changes in fermentations, such as nitrogen limitation (Monteiro and Bisson, 1991), low oxygen availability (Holm Hansen et al., 2001) and inhibition of increased ethanol (Fleet, 2003), as well as extrinsic factors such as SO_2_(Ribéreau-Gayon et al., 2006). However, Nissen et al. (2003) proposed that *S. cerevisiae* S101 adopted a contact-dependent mechanism to induce the culturability loss of some non-*Saccharomyces* strains (*Lachancea thermotolerans* and *Torulaspora delbrueckii*). Subsequently, the contact-dependent mechanism was confirmed by studies using the same *S. cerevisiae* strain (Nissen et al., 2004; Renault et al., 2013; Kemsawasd et al., 2015a). However, it was found that *S. cerevisiae* CCMI 885 excreted toxic compounds, which inhibited the growth of *Hanseniaspora guilliermondii* and *H. uvarum*, demonstrating the interaction of these species through excreted antimicrobial compounds (Pérez-Nevado et al., 2006). Recent studies further elucidated that *S. cerevisiae* CCMI 885 produced antimicrobial peptides, which altered intracellular pH, membrane permeability and culturability of non-*Saccharomyces* strains (Albergaria et al., 2010; Branco et al., 2014, 2015). Interestingly, in the work of Wang et al. (2015c), not only the excreted products from *S. cerevisiae* NSa but also the synthetic media, induce a lack of culturability of *H. uvarum*. However, the synthetic must was weaker at inducing a lack of culturability of *H. uvarum* than *S. cerevisiae* supernatant, which included the same media plus the yeast metabolites. Thus, the role of environmental changes should be taken into consideration when studying the interaction between different yeasts.

Until now, studies on culturability loss of non-*Saccharomyces* yeasts have mainly focused on several potential wine starters: *H. guilliermondii, H. uvarum, Kluyveromyces marxianus, L. thermotolerans*, and *T. delbrueckii* (reviewed in Albergaria and Arneborg, 2016). However, few studies have focused on the culturability differences among strains. According to Branco et al. (2014), different *D. bruxellensis* strains showed strain-specific sensitivity toward antimicrobial peptides excreted by *S. cerevisiae*. The differences between contact-dependent mechanisms and interactions through extracellular compounds were ascribed to the *S. cerevisiae* strains used (Kemsawasd et al., 2015a). Therefore, more yeast species and strains should be considered to gain a better understanding of the interaction between *S. cerevisiae* and non-*Saccharomyces* yeasts.

This study was aimed at (i) investigating the strain and species differences in culturability loss, (ii) analyzing the interaction mechanisms that exist in different strains, and (iii) determining the viable status of non-culturable cells. We investigated the interaction between *S. cerevisiae* NSa (the same strain used in our former work, Wang et al., 2015c) and 10 non-*Saccharomyces* strains from different sources belonging to *H. uvarum, S. bacillaris, M. pulcherrima*, and *T. delbrueckii* to analyze the interactions in mixed fermentation between *S. cerevisiae* and each individual strain. Through the use of three types of media (supernatants from *S. cerevisiae* fermentation, synthetic media mimicking *S. cerevisiae* fermentation and mixed fermentation), the performance of each non-*Saccharomyces* strain was compared and studied. Synthetic must was used to rule out other effects and to define the media to mimic the must at different stages of fermentation. Recovery analysis and viability assays were also conducted to evaluate the status of non-culturable cells.

## Materials and methods

### Yeasts strains and culture conditions

Eleven yeast strains were used in this study, containing *H. uvarum* CECT13130, NSb and CECT1444^T^, *S. bacillaris* NSc, NSd and CECT11046, *M. pulcherrima* Mp com and Mp 51, *T. delbrueckii* Td com and CECT13135, and *S. cerevisiae* NSa. These strains were obtained from different collections: CECT13130, NSa, NSb, NSc, NSd, Mp 51, CECT13135 and NSa were natural isolates from our collection (Wang et al., 2014). CECT1444 and CECT11046 were from Spanish Type Culture Collection. Mp com (Flavia) and Td com (Biodiva) were commercial strains from Lallemand Inc. (Canada).

The species identity of all strains was determined by 5.8S-ITS-RFLP analysis (Esteve-Zarzoso et al., 1999) and sequence analysis of the D1/D2 domain of 26S rDNA (Kurtzman and Robnett, 1998). Yeasts were grown overnight in YPD medium (1% yeast extract, 2% peptone, and 2% glucose, w/v, pH 6.2) at 28°C before use.

### Alcoholic fermentations, sampling, and setting culturability

Synthetic must (100 g/L fructose, 100 g/L glucose, 290 mg N/L amino nitrogen, and 120 mg N/L ammonium nitrogen, pH 3.3) was prepared according to Andorrà et al. (2012). 350 mL of synthetic must was added to a 500 mL screw cap bottle, inoculated with 10^6^ cells/mL of each yeast strain and kept at 25°C in a shaker at a speed of 120 rpm. Fermentations were performed in the presence of air because the caps were not screwed tightly on the bottles. Each of the mixed fermentations was inoculated with one non-*Saccharomyces* strain and *S. cerevisiae* NSa. As a comparison, a single *S. cerevisiae* fermentation was carried out with the NSa strain. Fermentations were conducted in duplicate; when an interaction was observed, the fermentations were repeated in another duplicate and thus four replicas were used to set the interaction analysis.

Samples were taken every day to follow sugar and nitrogen consumption, ethanol production and yeast population dynamics until the end of fermentation. Concentrations of ethanol, fructose and glucose were tested using an enzymatic kit from Roche Diagnostics (Germany). The level of individual amino acids and ammonium was analyzed by HPLC according to Andorrà et al. (2012). Yeast populations in all samples were quantified using a microscope and plating after appropriate dilution in sterile water. YPD agar medium was used to calculate the total number of yeast cells present, and lysine agar medium (Oxoid LTD., England) was used for quantification of non-*Saccharomyces* strains.

**Figure 1:**
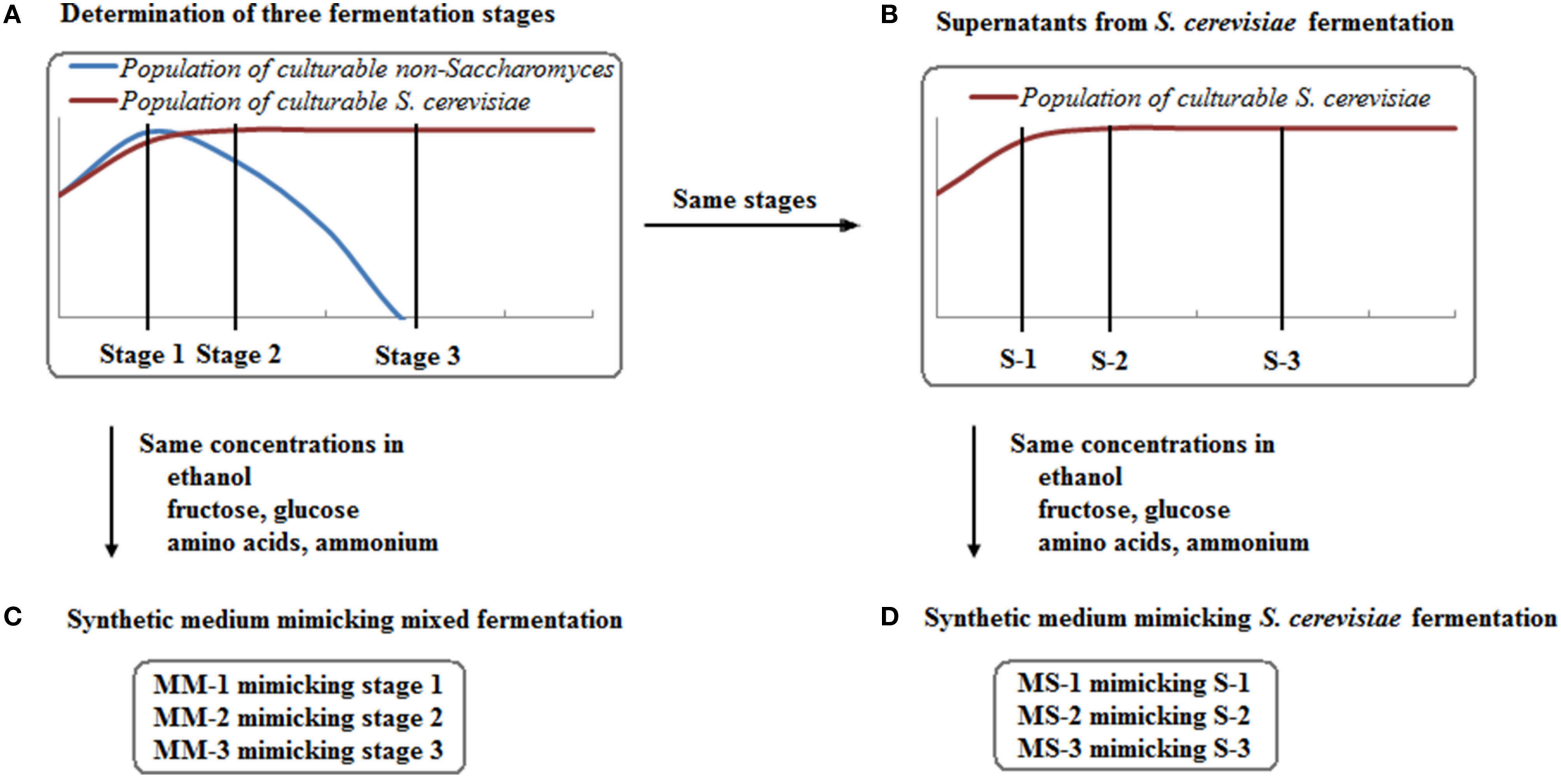
**Experimental design for setting up three fermentation stages (A) and preparing three types of synthetic media (B–D)**.

Three stages were set up for each species depending on the culturability of the non-*Saccharomyces* species in mixed fermentations (Figure 1A): 1. When culturable populations reached the highest level; 2. When culturable populations started to decrease; 3. When no colonies grew on plates, or at the end of fermentation for some strains if colonies were still seen on plates. The concentration of main chemical components (ethanol, fructose, glucose, individual amino acids, and ammonium), fermentation time and non-*Saccharomyces* strain culturability at these stages were listed to mimic the conditions of each fermentation stage where the interaction between non-*Saccharomyces* strains and *S. cerevisiae* was set (Figure 1 and Table 1). Data from these stages at mixed and single fermentations were used for the interaction assays in next step.

**Table 1:** **Fermentation stages, population size and chemical characteristics of the media at different single and mixed fermentation stages**.

**Species**	**Fermentation time (h)**	**Culturable non-*saccharomyces* (cfu/mL)**	**Ethanol (v/v)**	**Fructose (g/L)**	**Glucose (g/L)**	**Total assimilable nitrogen (mg N/L)**	**Names of synthetic media**
*H. uvarum*	24	2.6 ± 1.3 × 10^7^	1.6 ± 0.2	74.6 ± 4.3	59.0 ± 4.1	66.8 ± 26.7	MM-1
	24	–	1.8 ± 0.0	76.7 ± 0.0	52.3 ± 0.0	5.0 ± 0.0	MS-1
	48	2.0 ± 1.1 × 10^7^	3.1 ± 0.6	49.8 ± 2.1	25.1 ± 6.1	6.6 ± 3.3	MM-2
	48	–	4.2 ± 0.1	45.2 ± 1.6	13.7 ± 1.7	1.2 ± 0.0	MS-2
	96	nd	9.4 ± 0.5	12.7 ± 5.0	0.9 ± 0.7	nd	MM-3
	96	–	10.2 ± 0.4	4.1 ± 2.9	nd	nd	MS-3
*M. pulcherrima*	24	8.1 ± 3.3 × 10^6^	2.2 ± 0.6	89.6 ± 4.1	53.1 ± 7.2	87.4 ± 54.1	MM-1
	24	–	1.8 ± 0.0	76.7 ± 0.0	52.3 ± 0.0	5.0 ± 0.0	MS-1
	48	3.2 ± 6.8 × 10^6^	6.7 ± 0.9	56.1 ± 4.9	23.6 ± 5.7	2.5 ± 1.2	MM-2
	48	–	4.2 ± 0.1	45.2 ± 1.6	13.7 ± 1.7	1.2 ± 0.0	MS-2
	96	nd	10.2 ± 0.5	15.9 ± 6.0	0.7 ± 0.8	nd	MM-3
	96	–	10.2 ± 0.4	4.1 ± 2.9	nd	nd	MS-3
*S. bacillaris*	24	4.2 ± 3.7 × 10^7^	1.5 ± 0.2	68.6 ± 3.0	50.7 ± 2.2	18.3 ± 4.7	MM-1
	24	–	1.8 ± 0.0	76.7 ± 0.0	52.3 ± 0.0	5.0 ± 0.0	MS-1
	96	8.8 ± 8.3 × 10^6^	9.9 ± 0.5	5.1 ± 1.9	nd	nd	MM-2
	96	–	10.2 ± 0.4	4.1 ± 2.9	nd	nd	MS-2
	120	4.7 ± 8.2 × 10^1^	11.6 ± 0.1	0.1 ± 0.1	nd	nd	MM-3
	120	–	11.5 ± 0.4	0.1 ± 0.1	nd	nd	MS-3
*T. delbrueckii*	24	1.9 ± 0.7 × 10^7^	3.1 ± 0.1	90.8 ± 3.4	48.4 ± 0.9	20.1 ± 13.9	MM-1
	24	–	1.8 ± 0.0	76.7 ± 0.0	52.3 ± 0.0	5.0 ± 0.0	MS-1
	96	1.1 ± 1.9 × 10^6^	10.4 ± 0.4	19.8 ± 5.1	1.1 ± 1.9	nd	MM-2
	96	–	10.2 ± 0.4	4.1 ± 2.9	nd	nd	MS-2
	144	1.8 ± 2.4 × 10^6^	11.7 ± 0.6	1.5 ± 1.9	nd	nd	MM-3
	120	–	11.5 ± 0.4	0.1 ± 0.1	nd	nd	MS-3

*This chemical composition was used to define the media mimicking the three selected fermentation stages. All values are the average of different strains within the same species. “MM” means synthetic media with main metabolites (ethanol, fructose, glucose and nitrogen) mimicking mixed fermentations, whereas “MS” is named after synthetic media with main metabolites mimicking S. cerevisiae fermentation. The Arabic numbers 1, 2, and 3 refer to the three stages selected in fermentations. “–” refers to the absence of a non-Saccharomyces population as derived from single S. cerevisiae fermentations, whereas “nd” means not detected. The total assimilable nitrogen is the sum of nitrogen from assimilable amino acids and ammonium. Only amino acid concentrations higher than 0.9 mg N/L are considered, and the concentrations are shown in Supplementary Table 1*.

### Culture assays using different synthetic media

To further understand the culturability of non-*Saccharomyces* strains in mixed fermentation, three types of synthetic media were prepared (Figure 1): supernatant from *S. cerevisiae* fermentation (S), synthetic medium mimicking *S. cerevisiae* fermentation (MS) and synthetic medium mimicking mixed fermentation (MM). S was collected from *S. cerevisiae* fermentation (Figure 1B), centrifuged and filtered using a 0.22 μm Whatman syringe filter (GE Healthcare Life Science, Germany). S was spread onto YPD agar plates to confirm the absence of *S. cerevisiae* cells. As a comparison, MS was prepared with metabolites (ethanol, fructose, glucose, individual amino acid, and ammonium) mimicking S, with the absence of *S. cerevisiae* excreted compounds (Figure 1D). By performing culture assays using S and MS, the effect of main fermentation metabolites (the same for S and MS) and other putative *S. cerevisiae* metabolites (only in S) could be observed. Considering the possible differences of the main metabolites produced by *S. cerevisiae* fermentation and mixed fermentation, MM was prepared with corresponding components mimicking the mixed fermentation (Figure 1C). Moreover, no micronutrients or vitamins were added to MS and MM due to fast consumption at the beginning of alcoholic fermentation. All of the synthetic media were prepared for the three fermentation stages selected in 2.2 and were named with Arabic numbers to differentiate these stages (Figure 1 and Table 1).

Single fermentations of each non-*Saccharomyces* strain were then performed to provide adapted cells as described in Wang et al. (2015c). These adapted cells were incubated in YPD to ensure viability and incubated in synthetic media to check their culturability on plates. Culture assays were conducted at 25°C in duplicate with a shaking speed of 120 rpm; when a culturability decrease was observed, the culture assays were repeated in another duplicate and thus four replicas were used to follow the culturability changes of non-*Saccharomyces* yeasts. Samples were taken at 24, 48, and 120 h to quantify yeast cells using a microscope and YPD plating after appropriate dilution in sterile water. Cells losing culturability in synthetic media were collected for the following recovery analysis and viability assay.

### Recovery analysis and viability assay of non-culturable cells from synthetic media

To test the viability of non-culturable cells from synthetic media, two approaches were used. Membrane integrity was analyzed by using the LIVE/DEAD® BactLight™ Bacterial Viability kit (Molecular Probes Inc., USA). In this assay, yeast cells were stained and observed using a fluorescence microscope equipped with filter system I3 and N2.1 (Leica DM 4000B) as in Hierro et al. (2006). The capacity to grow in rich liquid media was analyzed by incubating the cells in fresh YPD medium. Cells that could be recovered were considered to be viable but not culturable in synthetic media. Cells that could not be recovered after two consecutive 48 h incubations in fresh YPD medium were analyzed again by the LIVE/DEAD® BactLight™ Bacterial Viability kit.

### Statistical analysis

One-way ANOVA by IBM SPSS Statistics 23 was used to calculate the value of significance for the variation analysis, and included a *post-hoc* Tukey test when needed. The consumption ratio (% of the total) of nutrients was used directly for the analysis of variation.

## Results

### Culturable population and metabolic characteristics of non-*saccharomyces* strains during alcoholic fermentation

**Figure 2:**
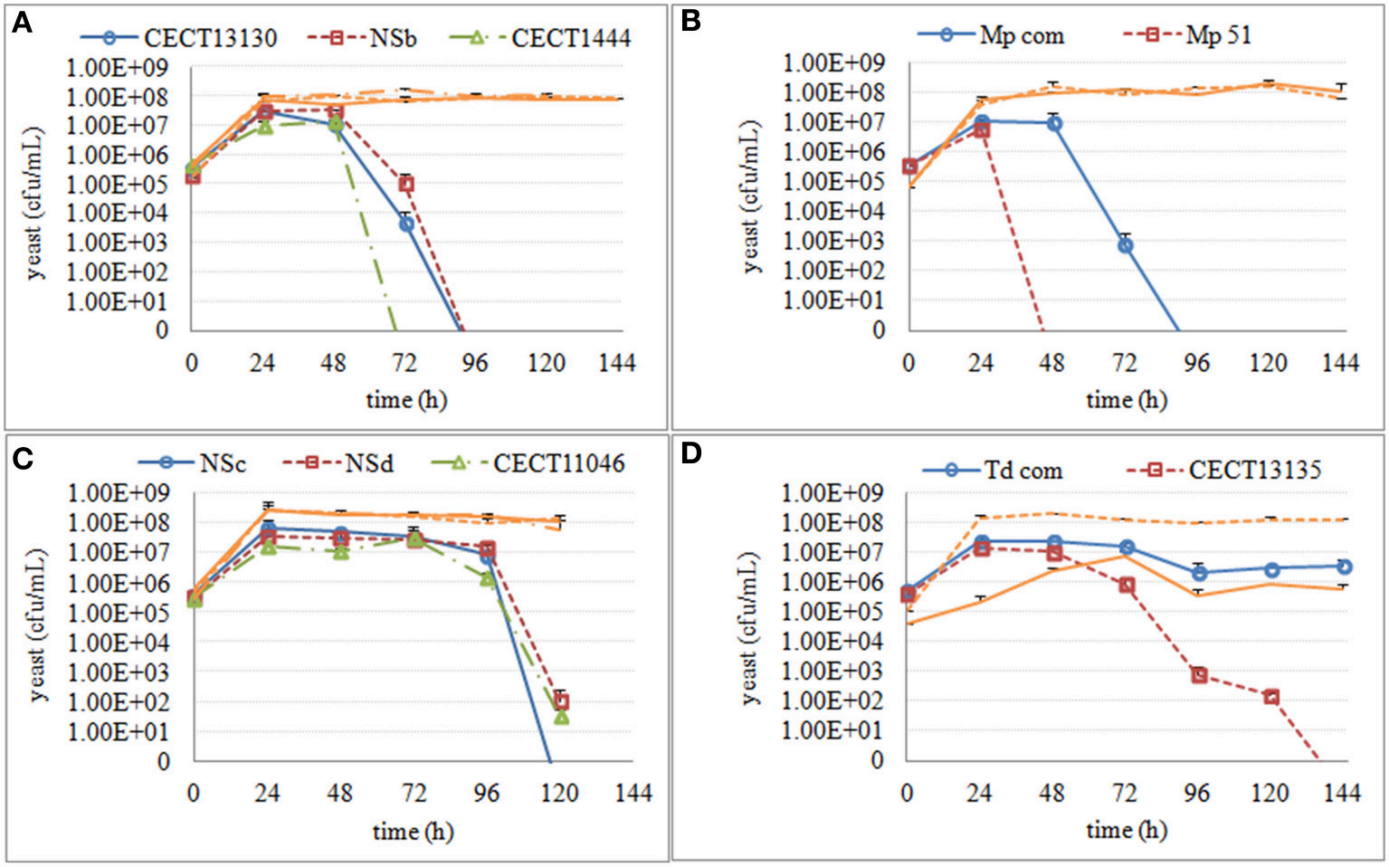
**Culturable population of non-***Saccharomyces*** in mixed fermentations with ***S***. ***cerevisiae*****. Culturable *S. cerevisiae* populations were shown in orange line using the same line type as the non-*Saccharomyces* co-inoculated. **(A)** *H. uvarum* **(B)** *M. pulcherrima* **(C)** *S. bacillaris* **(D)** *T. delbrueckii*.

Overall, both *S. cerevisiae* and non-*Saccharomyces* strains reached the maximum population number of 10^7^–10^8^ cells/mL 24 h after inoculation, and this size was maintained during mixed and single fermentations. Culturability of non-*Saccharomyces* strains decreased in all mixed fermentations. This decrease varied not only among different yeast species but also among some strains within the same species (Figure 2). Culturable *H. uvarum* increased to 10^7^–10^8^ cfu/mL at 24 h and began to decrease at 48 h. No colonies were formed on lysine plates for CECT1444 after 72 h and for CECT13130 and NSb after 96 h. Similar to *H. uvarum, M. pulcherrima* grew to 10^7^ cfu/mL and quickly started to decrease. At 96 h, no colonies of Mp com were recovered on lysine plates, and for Mp 51, no colonies were recovered after 48 h. Culturable *S. bacillaris* maintained a population size of 10^7^–10^8^ cfu/mL until 96 h at which time the population started to decline. After 120 h, 10 to 100 cfu/mL of NSd and CECT11046 were recovered, however no colonies of NSc grew from lysine plates. Finally, *T. delbrueckii* strains reached approximately 10^7^ cfu/mL and were maintained up to 48 h. After 48 h, CECT13135 started to decline, and no colonies were recovered after 144 h. The other *T. delbrueckii* strain, Td com, showed a slow decrease in culturable population, and at 144 h, 10^6^ cfu/mL of Td com were still culturable. Based on the culturability of non-*Saccharomyces* strains in mixed fermentations, three stages were set up for each species. The fermentation times and main metabolites of these three fermentation stages are shown in Table 1. As a comparison, the same stage in fermentation of *S. cerevisiae* is also listed.

Despite the different culturability of non-*Saccharomyces* strains in mixed fermentations, no obvious variations in fermentation length were observed, and all fermentations finished after 120 or 144 h. Similar to *S. cerevisiae* fermentation, all mixed fermentations consumed glucose faster than fructose, and the final ethanol concentration reached 11–12% V (Table 1). However, analysis of the consumption of these main metabolites after 24 h revealed strain-dependent differences. As shown in Table 2, the strains that lost culturability faster were those that consumed some metabolites faster in the mixed fermentations: (i) Within *H. uvarum*, mixed fermentations inoculated with CECT1444 consumed glucose, ammonium and arginine faster than the other two strains; (ii) For *M. pulcherrima*, mixed fermentations with Mp 51 metabolized fructose, glucose, alanine, ammonium, arginine and glutamine faster than mixed fermentations with Mp com; (iii) Arginine was consumed faster during mixed fermentations with *S. bacillaris* NSc than the other two strains; (iv) More arginine was consumed during mixed fermentations with *T. delbrueckii* CECT13135 than Td com.

**Table 2:** **Consumption ratio of glucose, alanine, ammonium, arginine, and glutamine at 24 h of fermentation**.

	**Non-*Saccharomyces* strains**	**% of the total**						**Time of culturability loss**
		**Fructose**	**Glucose**	**Alanine**	**Ammonium**	**Arginine**	**Glutamine**	
sc		23.3	47.7	100.0	100.0	100.0	100.0	nd
sc+hu	CECT13130	27.2	37.6^*^	90.4^*^	68.1^*^	65.1^*^	100.0	96 h
	NSb	23.3	39.2^*^	93.1^*^	63.4^*^	66.7^*^	100.0	96 h
	CECT1444	25.7	46.2^#^	91.6^*^	100.0^#^	71.9^*^^#^	100.0	72 h
sc+mp	Mp com	6.9^*^^#^	40.7^*^^#^	13.4^*^^#^	69.1^*^^#^	53.7^*^^#^	88.4^*^^#^	96 h
	Mp 51	13.9^*^	53.2^*^	96.7	91.2^*^	67.8^*^	100.0	48 h
sc+sb	NSc	35.1^*^	51.6	100.0	100.0	91.4^*^^#^	100.0	120 h
	NSd	30.4^*^	48.5	96.9	100.0	82.7^*^	100.0	nd
	CECT11046	28.8^*^	47.7	98.3	100.0	84.1^*^	100.0	nd
sc+td	Td com	10.8^*^	51.6	99.4	100.0	75.9^*^^#^	100.0	nd
	CECT13135	7.7^*^	51.6	99.9	100.0	93.5^*^	100.0	144 h

*sc means S. cerevisiae single fermentation, and mixed fermentations are presented as sc+hu (S. cerevisiae + H. uvarum), sc+mp (S. cerevisiae + M. pulcherrima), sc+sb (S. cerevisiae + S. bacillaris), sc+td (S. cerevisiae + T. delbrueckii). nd means not detected*.

* *significance ≤ 0.05 with respect control (sc) by one-way ANOVA*.

# *significantly different from the other strains of the same species as determined by a post-hoc Tukey test*.

### The influence of excreted compounds from *S. cerevisiae* and media composition on the culturability of non-*saccharomyces* strains

To further elucidate the culturability of non-*Saccharomyces* strains and the interaction with *S. cerevisiae* during mixed fermentation, we performed culture assays using S (supernatant from *S. cerevisiae* fermentation), MS (synthetic media mimicking *S. cerevisiae* fermentation), and MM (synthetic media mimicking mixed fermentation) based on the three stages of fermentation (Table 1). Although the non-*Saccharomyces* strains maintained a population size of 10^7^–10^8^ cells/mL for 120 h, as determined by cell counting under a microscope, not all strains were culturable during the 5-day period. The culturability was dependent on the media used, as well as the yeast species and strain.

**Figure 3:**
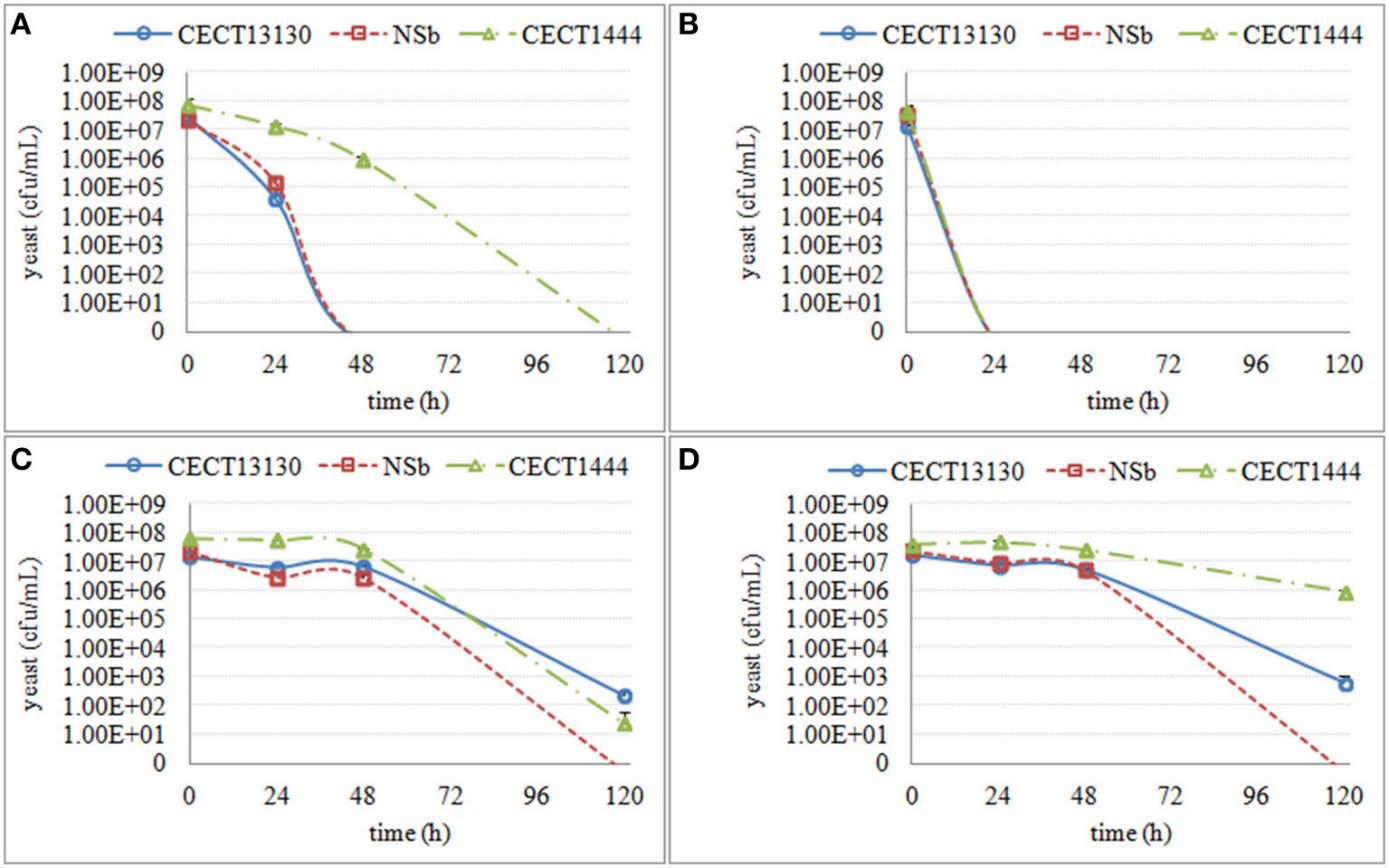
**The culturable population of three ***H. uvarum*** strains (CECT13130, NSb and CECT1444) grown in different synthetic media for 120 h**. **(A)** the growth in supernatant from the second stage of *S. cerevisiae* fermentation S-2 **(B)** the growth in supernatant from the third stage of *S. cerevisiae* fermentation S-3 **(C)** the growth in synthetic media MS-3 **(D)** the growth in synthetic media MM-3.

**Figure 4:**
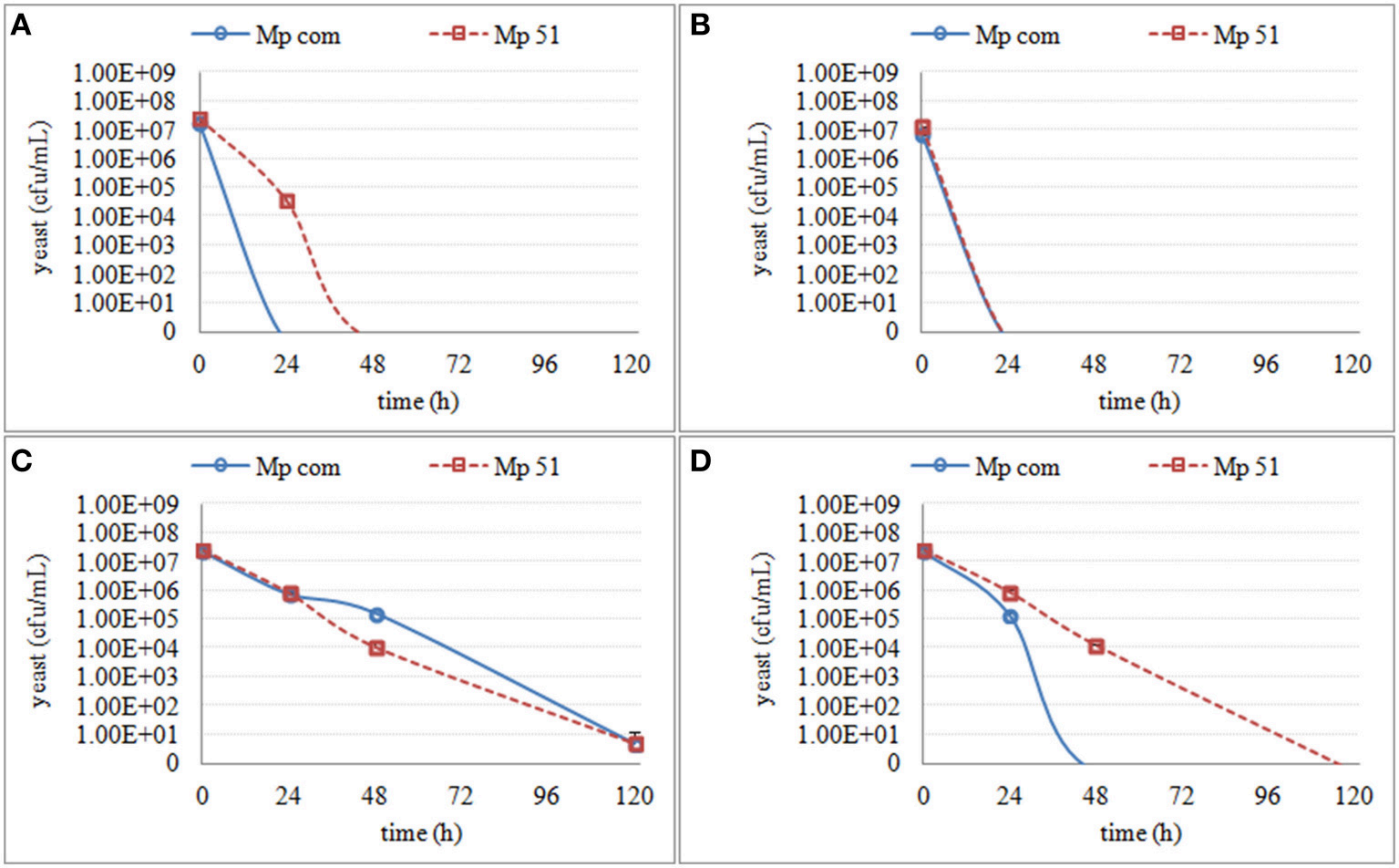
**The culturable population of two ***M. pulcherrima*** strains (Mp com and Mp 51) grown in different synthetic media for 120 h**. **(A)** the growth in supernatant from the second stage of *S. cerevisiae* fermentation S-2 **(B)** the growth in supernatant from the third stage of *S. cerevisiae* fermentation S-3 **(C)** the growth in synthetic media MS-3 **(D)** the growth in synthetic media MM-3.

**Figure 5:**
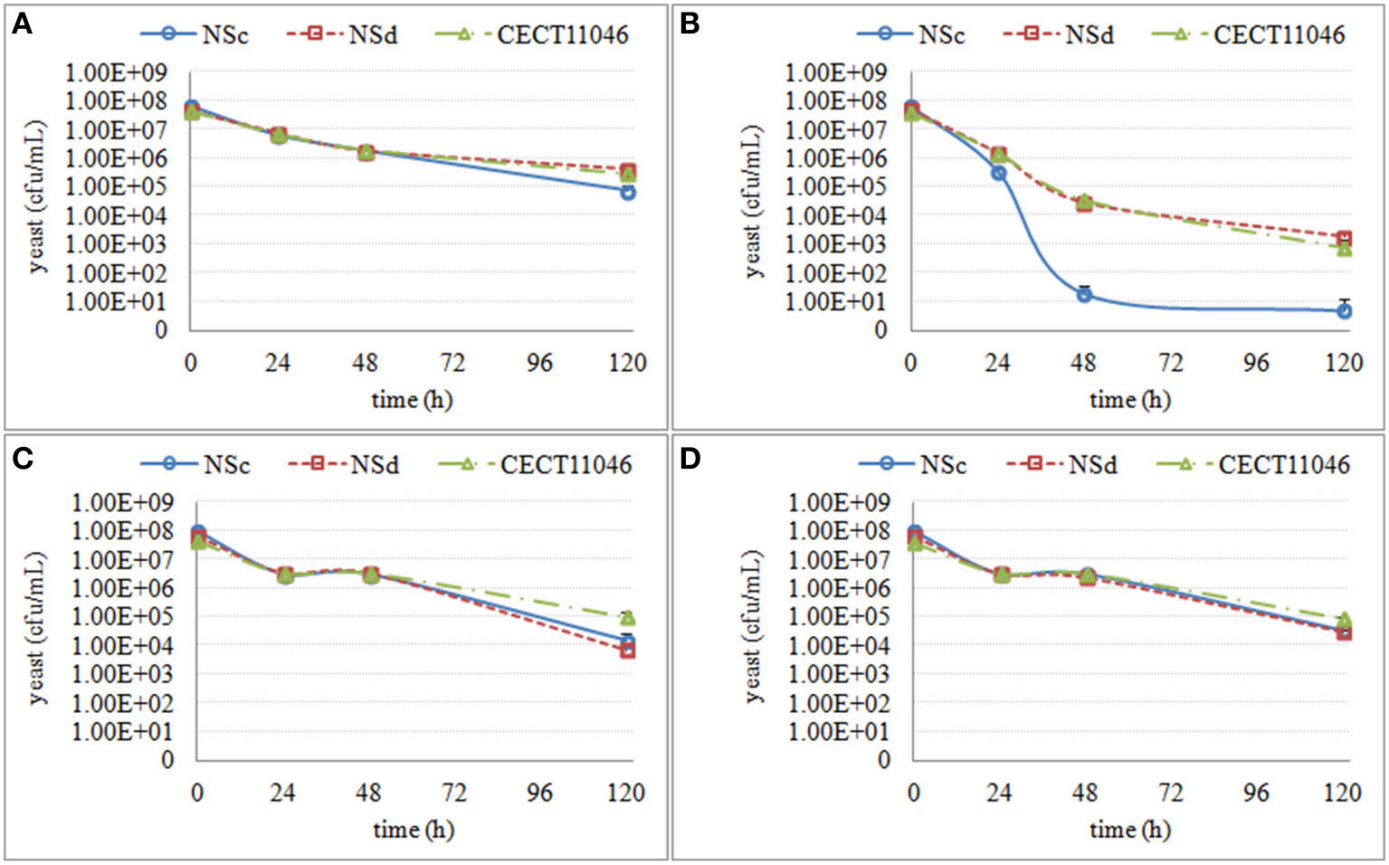
**The culturable population of three ***S. bacillaris*** strains (NSc, NSd and CECT11046) grown in different synthetic media for 120 h**. **(A)** the growth in supernatant from the second stage of *S. cerevisiae* fermentation S-2 **(B)** the growth in supernatant from the third stage of *S. cerevisiae* fermentation S-3 **(C)** the growth in synthetic media MS-3 **(D)** the growth in synthetic media MM-3.

**Figure 6:**
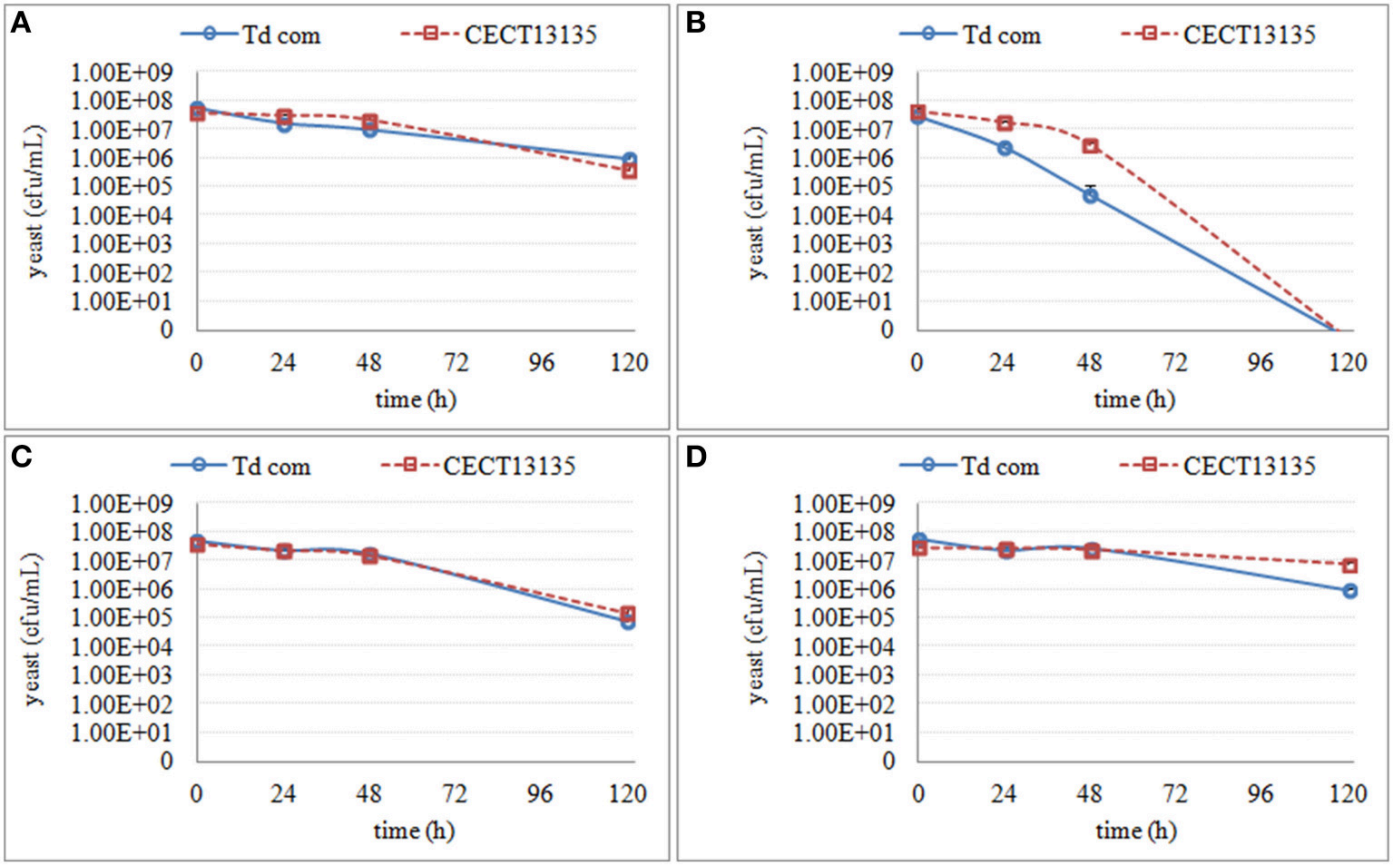
**The culturable population of two ***T. delbrueckii*** strains (Td com and CECT13135) grown in different synthetic media for 120 h**. **(A)** the growth in supernatant from the second stage of *S. cerevisiae* fermentation S-2 **(B)** the growth in supernatant from the third stage of *S. cerevisiae* fermentation S-3 **(C)** the growth in synthetic media MS-3 **(D)** the growth in synthetic media MM-3.

No effect on culturability was seen in any media in fermentation stage 1 (S-1, MS-1, MM-1), which corresponded to the fermentation stage where culturable populations of non-*Saccharomyces* strains were the highest (generally between 10^7^ and 10^8^ cfu/ml). However, in the media from fermentation stage 2 (only S-2), and in the media from fermentation stage 3 (all the media), a decrease in culturable populations was observed (Figures 3–**6**), although the extent of the decrease was species- and strain-dependent.

Within *H. uvarum* strains, the decrease of culturability was seen at 24 h in S-2 and S-3 and at 120 h in MS-3 and MM-3 (Figure 3). *H. uvarum* strains lost culturability in both S-2 and S-3; however, the decrease in culturability in S-2 occurred more slowly than in S-3. Further, among the three strains, CECT1444 showed a much slower decrease in culturability in S-2. The media (MS-3 and MM-3) also affected the culturability, but to a lesser extent, and they were evident only at 120 h. There was also a strain difference, such that NSb was more affected than the other two strains.

Regarding the *M. pulcherrima* strains, a slow decrease in culturability was observed in all media mimicking the fermentation stages (MS-2, MM-2, MS-3, and MM-3), whereas a sharp decrease was seen in the *S. cerevisiae* supernatants (S-2 and S-3). Mp com and Mp51 showed different culturability in both S-2 and MS-3 (Figure 4), with Mp com exhibiting higher sensitivity.

The culturability of three *S. bacillaris* strains was less affected by different synthetic media, as some colonies were recovered (Figure 5). All of the strains showed a decrease in culturability during all studied periods (120 h), with no relevant differences between strains. Only in S-3 was a difference in sensitivity observed with strain NSc, which showed much lower culturability than the other two strains.

Similar to *S. bacillaris*, the effect of synthetic media on the culturability of the two *T. delbrueckii* strains was limited (Figure 6). However, when the cells were cultured in S-3, no colonies were recovered after 120 h. Instead, only a small decrease of culturability was observed in MS-3 and S-2.

Although the decrease of culturability varied among different species, S-3 consistently showed the most obvious effect compared with other synthetic media. For the two species more affected (*H. uvarum* and *M. pulcherrima*), a more obvious effect was shown in S-2 than in MS-3 or MM-3. Thus, it is likely that some substances secreted from *S. cerevisiae* played a principal role in the interaction between *S. cerevisiae* and non-*Saccharomyces* strains, and that changes in the media (ethanol, nitrogen and sugar) also mediated the interaction.

### The viability of the non-culturable cells

To improve the understanding of the lack of culturability of the non-*Saccharomyces* strains, all of the samples (34 cases in total) that did not grow on plates were tested using two different methods: membrane integrity using the LIVE/DEAD viability kit and recovery by suspension in liquid YPD with agitation.

On one hand, these non-culturable cells were immediately analyzed using the LIVE/DEAD viability kit (Supplementary Figure 1). The results showed that live fluorescent cells were only found three times: non-culturable cells of the Mp com strain at 24 h in S-2 yielded 0.13% of live fluorescent cells, at 48 h in MM-3 8.17% and the Mp 51 strain at 120 h in MM-2 2.84 %. All other non-culturable cells yielded dead fluorescent cells.

On the other hand, all non-culturable cells were evaluated by recovery analysis. The non-culturable cells with live fluorescent were recovered when incubated in liquid YPD medium, whereas some of the non-culturable cells with dead fluorescent could also be recovered. The latter cases were found seven times involving three non-*Saccharomyces* species. For example, after 120 h exposure to mimicking media MS-3 and MM-3, *H. uvarum* NSb, as well as *H. uvarum* CECT1444 after S-2 and the two *T. delbrueckii* strains after S-3, appeared dead in the fluorescence analysis but could be recovered. In the case of *H. uvarum* CECT13130, this observation was seen only in the early stages of exposure (48 h) to the media. Likewise, Mp com could be recovered after 120 h exposure to mimicking media MM-2 but could not be recovered from MM-3. Both cases indicate the existence of an intermediate, transient step before the cells are completely dead.

The LIVE/DEAD viability kit was again used to check the cells that could not be recovered by consecutive incubation in liquid YPD medium. All cells that could not be recovered yielded only dead fluorescent.

## Discussion

The culturability loss of non-*Saccharomyces* strains during late stages of alcoholic fermentation has been well documented (Fleet, 2003). However, despite recent advances, the cellular mechanism underlying culturability loss is still a matter of discussion (Ciani and Comitini, 2015; Liu et al., 2015; Albergaria and Arneborg, 2016). In a previous study (Wang et al., 2015c), we investigated how *S. cerevisiae* NSa interacted with *H. uvarum* NSb by the use of a compartmented dialysis system, cell-free supernatant and mimicking synthetic media. Due to the absence of a contact-dependent mechanism in *S. cerevisiae* NSa, in the present study we decided to focus on the effects of compounds secreted by *S. cerevisiae* NSa, and the changes in main metabolites (ethanol, glucose, fructose, amino nitrogen and ammonium nitrogen).

Our results indicated that cell-free supernatant from *S. cerevisiae* fermentation influenced cellular culturability much more than mimicking synthetic media at the same fermentation stage (same chemical composition for major metabolites). Therefore, as mentioned in Wang et al. (2015c), some putative *S. cerevisiae* metabolites played a main role in the interaction between *S. cerevisiae* NSa and other non-*Saccharomyces* strains. A faster culturability loss was induced by *S. cerevisiae* supernatant at stage 3 than the initial two stages, which demonstrated the possible accumulation, or higher effect, of the *S. cerevisiae* secreted compounds as fermentation proceeded. Studies from Pérez-Nevado et al. (2006) and Williams et al. (2015) further related the accumulation to the amount of sugar consumed by *S. cerevisiae*. Likewise, antimicrobial peptides identified by Branco et al. (2014) were derived from a glycolytic enzyme, showing a probable link with sugar metabolism of *S. cerevisiae*. More research is still required to illustrate how sugar consumption regulates the secretion of antimicrobial peptides or other putative metabolites.

Moreover, according to our previous report (Wang et al., 2015c), as fermentation proceeds, the changes of the main metabolites also decreased the culturability of the cells, and the present results indeed showed that synthetic media at stage 3 caused a decrease in culturability. However, this effect occurs more slowly in the sensitive species and strains, and in our culture assays, complete culturability loss was mostly found after 48 or 120 h. This indicates that the changes in the main metabolites play a role in the interaction between *S. cerevisiae* NSa and other non-*Saccharomyces* strains, and vice versa. Because not all of the species were equally affected, it also showed the capacity of some non-*Saccharomyces* strains to withstand a harsh environment (ethanol higher than 10% vol, glucose lower than 1 g/L, fructose lower than 16 g/L and no available nitrogen).

The interaction between *S. cerevisiae* and non-*Saccharomyces* strains also relied on the participating yeast species. In our mixed fermentations, cells of *S. bacillaris* and *T. delbrueckii* could coexist longer with *S. cerevisiae* than *H. uvarum* and *M. pulcherrima*. Other studies proposed that oxygen availability, glucose uptake rate and nitrogen source might contribute to the longer co-existence (Holm Hansen et al., 2001; Nissen et al., 2004; Andorrà et al., 2012; Taillandier et al., 2014). We indeed found that mixed fermentation inoculated with *S. bacillaris* or *T. delbrueckii* present a consumption rate of glucose, alanine, ammonium and arginine more similar to single fermentations with *S. cerevisiae* than those mixed fermentations inoculated with *H. uvarum* and *M. pulcherrima*. However, the relation between species tolerance and consumption of some nutrients still needs further investigation.

As expected, strain differences within each species were observed in mixed fermentation, culture assays and recovery analyses. First, strains decrease their culturability to a different extent during mixed fermentation. Second, when incubated in the same synthetic media in culture assays, strains showed different culturability or tolerance to a harsh environment. Third, non-culturable cells from the same synthetic media showed different recovery abilities depending on the strain. The strain difference, to some extent, increased the complexity of interaction analysis between *S. cerevisiae* and non-*Saccharomyces* strains. In our case, the hypothesis of “strain tolerance to hard environment” cannot simply explain the strain differences in mixed fermentations. *H. uvarum* CECT1444 exhibited a slow culturability decrease in S-2, MM-2 and MM-3 as compared to the other two strains and thus was regarded as a strain that is highly tolerant to harsh environments. However, despite being a highly tolerant strain, in mixed fermentation, the culturability decreased even earlier than the other two *H. uvarum* strains. When we analyzed the metabolites at 24 h of mixed fermentation inoculated with *H. uvarum* CECT1444, a faster consumption of glucose, ammonium and arginine was detected. Andorrà et al. (2012) and Kemsawasd et al. (2015b) reported the influence of nitrogen consumption on yeast growth and fermentation performance. Nevertheless, further research should be undertaken to elucidate this effect, which was also observed in strains Mp 51, *S. bacillaris* NSc and *T. delbrueckii* CECT13135 compared with other strains within the same species.

Another important finding was the appearance of non-culturable cells when incubated with synthetic media, yielding more than 90 % of cells with “dead” fluorescence by viability analysis but that could be recovered by incubation in YPD medium. However, when these cells were incubated longer in the synthetic media (24 h more), all showed dead fluorescence and could no longer be recovered in YPD medium. This phenomenon demonstrated the existence of VBNC status of at least *H. uvarum, M. pulcherrima*, and *T. delbrueckii* during alcoholic fermentation. As hypothesized by Branco et al. (2015), VBNC status could be understood as a transition status of yeast from culturable cells to dead cells, involving sub-lethally and severely injured cells. During this transition process, the ability to form colonies is the first lost vital activity and progressive changes in the permeability of cell membrane occur as found in this study, however the DNA or RNA remains stable (Andorrà et al., 2010; Wang et al., 2014, 2015a). Branco et al. (2015) measured how antimicrobial peptides secreted by *S. cerevisiae* affected cell viability and reported that injured cells had a similar pH as the external pH, whereas cells without compromised membranes (impermeable to propidium iodide) maintained a higher pH. Further research is still required to determine how the interactions between *S. cerevisiae* and non-*Saccharomyces* impacts physiological status and metabolic capacity of cells in different status.

In conclusion, we investigated the interaction between one *S. cerevisiae* strain and ten non-*Saccharomyces* strains during alcoholic fermentation. We demonstrated that the decrease of culturability was mainly caused by metabolites secreted by *S. cerevisiae*, although the change of the composition in main metabolites in the media also played a role. We also found that culturability loss of non-*Saccharomyces* yeasts was not only species-dependent but also strain-dependent. The finding of VBNC status and strain differences in culturability is meaningful to the exploration of *Saccharomyces*-non-*Saccharomyces* interactions. The understanding of these interactions is relevant for the development of non-*Saccharomyces* strains as starters in wine production.

## Author contributions

Conceived and designed the experiments: AM, BE. Performed the experiments: CW. Generated and analyzed the data: CW, AM, BE. Wrote the paper: CW, AM, BE.

### Conflict of interest statement

The authors declare that the research was conducted in the absence of any commercial or financial relationships that could be construed as a potential conflict of interest.
