# Supplementary material for: The Interaction between Saccharomyces cerevisiae and Non-Saccharomyces Yeast during Alcoholic Fermentation Is Species and Strain Specific
Source: Front Microbiol. 2016 Apr 13;7:502. doi: 10.3389/fmicb.2016.00502 (PMC4829597; doi:10.3389/fmicb.2016.00502)
Supplement: Supplementary file 1 [file DataSheet1.DOCX]

Supplementary Material

The Interaction between *Saccharomyces* *cerevisiae* and Non-*Saccharomyces* Yeast during Alcoholic Fermentation is Species and Strain Specific

Chunxiao Wang, Albert Mas^*^, Braulio Esteve-Zarzoso

**^*^Correspondence:** Albert Mas: [albert.mas@urv.cat](mailto:albert.mas@urv.cat)

# Supplementary Figures and Tables

## Supplementary Figure

| (**A**) | (**B**) | (**C**) |
| --- | --- | --- |
| 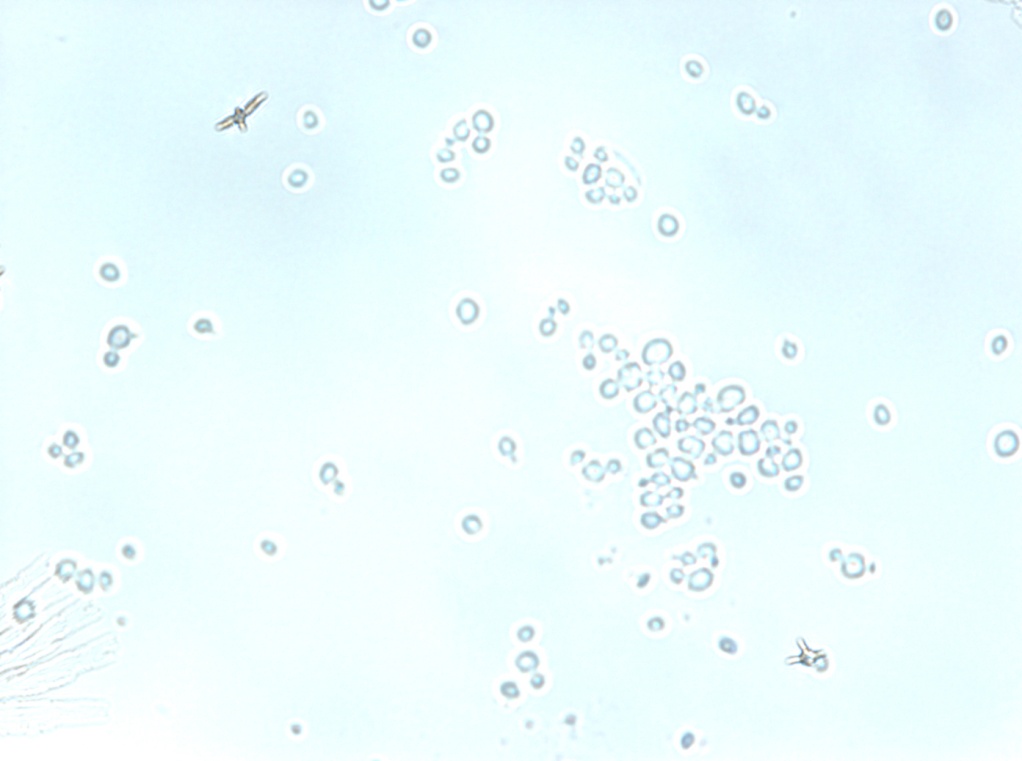 | 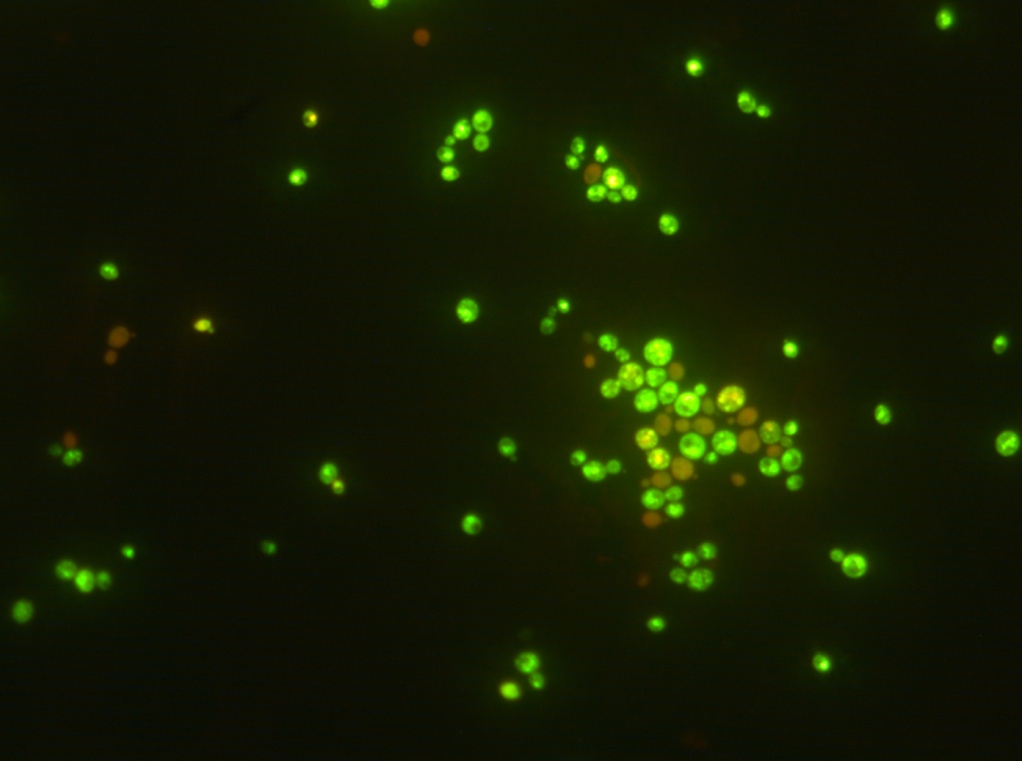  **L**  **D** | 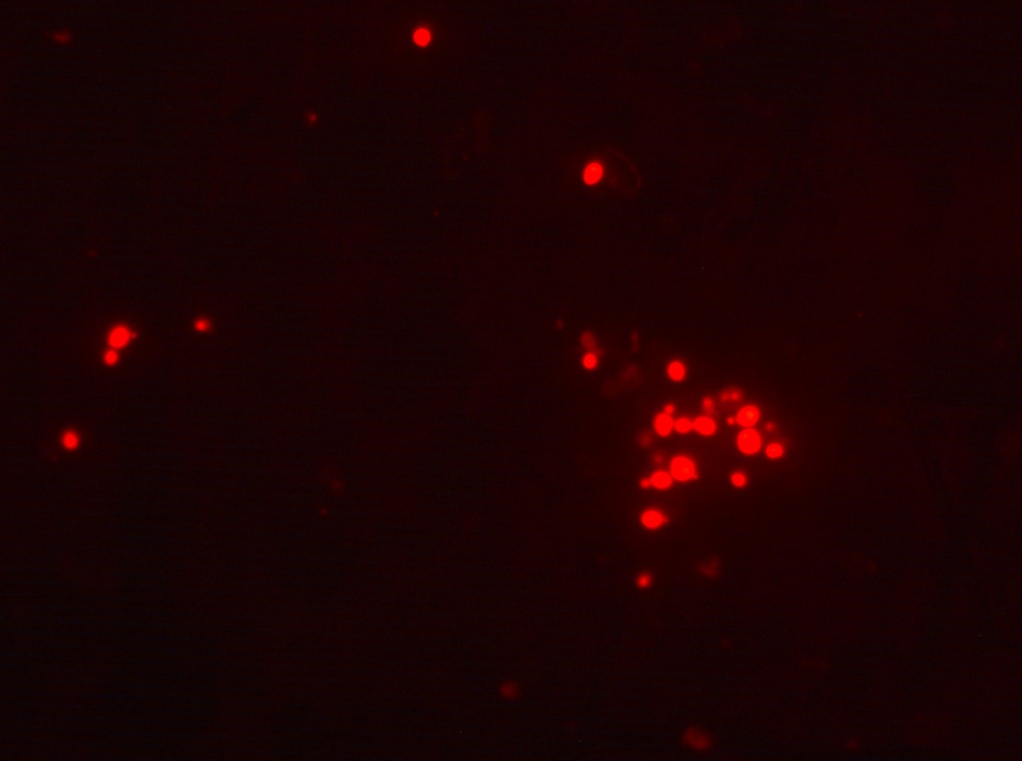  **D** |

**Supplementary Figure 1.** The cells with “L” (live) and “D” (dead) fluorescence in the viability assay. Cells were observed with white light (**A**), filter system I3 (**B**) and filter system N2.1 (**C**).

## Supplementary Table

**Supplementary Table 1.** The concentration of amino acids and ammonium in three selected stages of mixed fermentations inoculated with *S. cerevisiae* and one non-*Saccharomyces* species, and single fermentation with *S. cerevisiae*. All values are the average of different strains within the same species. Synthetic medium mimicking mixed fermentations is named MM, and synthetic medium mimicking *S. cerevisiae* fermentation is named MS. The Arabic numbers 1, 2 and 3 used in the name of synthetic media stand for the three stages selected in the fermentations. “nd” means not detected or the concentration is lower than 0.9 mg N/L. Synthetic media with only proline (30 mg N/L) is not listed.

| Species | Synthetic media | Alanine | Ammonium | Arginine | Aspartic | Cysteine | Glycine | Glutamic | Glutamine | Leucine | Proline | Tryptophane |
| --- | --- | --- | --- | --- | --- | --- | --- | --- | --- | --- | --- | --- |
|  |  | (mg N/L) | | | | | | | | | | |
| *H. uvarum* | MM-1 | 1.9 | 27.5 | 28.7 | 1.7 | 1.2 | 1.5 | nd | nd | 1.5 | 30.0 | 2.8 |
|  | MS-1 | nd | nd | nd | 1.3 | 1.1 | nd | nd | nd | 1.7 | 30.0 | 0.9 |
|  | MM-2 | nd | nd | 3.9 | 1.5 | nd | nd | nd | nd | nd | 30.0 | 1.2 |
|  | MS-2 | nd | nd | nd | nd | nd | nd | nd | nd | nd | 30.0 | 1.2 |
| *M. pulcherrima* | MM-1 | 10.4 | 23.9 | 35.1 | 2.1 | 1.0 | 2.2 | 1.6 | 5.6 | 0.5 | 30.0 | 5.1 |
|  | MS-1 | nd | nd | nd | 1.3 | 1.1 | nd | nd | nd | 1.7 | 30.0 | 0.9 |
|  | MM-2 | nd | nd | nd | 1.6 | nd | nd | nd | nd | 0.9 | 30.0 | nd |
|  | MS-2 | nd | nd | nd | nd | nd | nd | nd | nd | nd | 30.0 | 1.2 |
| *S. bacillaris* | MM-1 | nd | nd | 12.5 | 1.6 | 2.0 | nd | nd | nd | 1.0 | 30.0 | 1.4 |
|  | MS-1 | nd | nd | nd | 1.3 | 1.1 | nd | nd | nd | 1.7 | 30.0 | 0.9 |
| *T. delbrueckii* | MM-1 | nd | nd | 13.7 | 1.5 | 1.1 | nd | nd | nd | nd | 30.0 | 3.8 |
|  | MS-1 | nd | nd | nd | 1.3 | 1.1 | nd | nd | nd | 1.7 | 30.0 | 0.9 |
